# Booster Vaccination with BNT162b2 Improves Cellular and Humoral Immune Response in the Pediatric Population Immunized with CoronaVac

**DOI:** 10.3390/vaccines12080919

**Published:** 2024-08-15

**Authors:** Diego A. Díaz-Dinamarca, Simone Cárdenas-Cáceres, Nicolás A. Muena, Pablo Díaz, Gisselle Barra, Rodrigo Puentes, Daniel F. Escobar, Michal Díaz-Samirin, Natalia T. Santis-Alay, Cecilia Canales, Janepsy Díaz, Heriberto E. García-Escorza, Alba Grifoni, Alessandro Sette, Nicole D. Tischler, Abel E. Vasquez

**Affiliations:** 1 Subdepartamento Innovación y Desarrollo, Departamento Agencia Nacional de Dispositivos Médicos, Innovación y Desarrollo, Instituto de Salud Pública de Chile, Santiago 7780050, Chile; dadiaz@ispch.cl (D.A.D.-D.); pdiaz@ispch.cl (P.D.); gbarra@ispch.cl (G.B.); descobar@ispch.cl (D.F.E.);; 2 Laboratorio de Virología Molecular, Centro Ciencia & Vida, Fundación Ciencia & Vida, Santiago 8581151, Chile; scardenas@cienciavida.org (S.C.-C.); nmuena@cienciavida.org (N.A.M.); 3 Departamento Agencia Nacional de Dispositivos Médicos, Innovación y Desarrollo, Instituto de Salud Pública de Chile, Santiago 7780050, Chile; rpuentes@ispch.cl (R.P.); asantis@ispch.cl (N.T.S.-A.); ccanales@ispch.cl (C.C.); jdiaz@ispch.cl (J.D.); 4 Instituto de Salud Pública de Chile, Santiago 7780050, Chile; hgarcia@ispch.cl; 5 Center for Vaccine Innovation, La Jolla Institute for Immunology (LJI), La Jolla, CA 92037, USA; agrifoni@lji.org (A.G.); alex@lji.org (A.S.); 6 Department of Pathology, University of California San Diego, La Jolla, CA 92093, USA; 7 Escuela de Bioquímica, Facultad de Medicina y Ciencia, Universidad San Sebastián, Santiago 7510157, Chile

**Keywords:** SARS-CoV-2, SARS-CoV-2 neutralizing antibodies, COVID-19, BNT162b2 (Pfizer-BioNTech), CoronaVac, heterologous vaccination, Chile

## Abstract

The SARS-CoV-2 Omicron variant and its sublineages continue to cause COVID-19-associated pediatric hospitalizations, severe disease, and death globally. BNT162b2 and CoronaVac are the main vaccines used in Chile. Much less is known about the Wuhan-Hu-1 strain-based vaccines in the pediatric population compared to adults. Given the worldwide need for booster vaccinations to stimulate the immune response against new Omicron variants of SARS-CoV-2, we characterized the humoral and cellular immune response against Omicron variant BA.1 in a pediatric cohort aged 10 to 16 years who received heterologous vaccination based on two doses of CoronaVac, two doses of CoronaVac (2x) plus one booster dose of BNT162b2 [CoronaVac(2x) + BNT162b2 (1x)], two doses of CoronaVac plus two booster doses of BNT162b2 [CoronaVac(2x) + BNT162b2 (2x)], and three doses of BNT162b2. We observed that the [CoronaVac(2x) + BNT162b2 (2x)] vaccination showed higher anti-S1 and neutralizing antibody titers and CD4 and CD8 T cell immunity specific to the Omicron variant compared to immunization with two doses of CoronaVac alone. Furthermore, from all groups tested, immunity against Omicron was highest in individuals who received three doses of BNT162b2. We conclude that booster vaccination with BNT162b2, compared to two doses of CoronaVac alone, induces a greater protective immunity.

## 1. Introduction

Severe acute respiratory syndrome coronavirus type 2 (SARS-CoV-2) is a positive-stranded ssRNA(+) virus that has led to the severe coronavirus disease 2019 (COVID-19) pandemic that claimed more than seven million lives worldwide [1]. In Chile, a total of 62,249 deaths including confirmed, suspected, and probable cases have been reported, nearly all among adults [2]. The clinical manifestations of SARS-CoV-2 primary infections in children and adolescents are in general less severe compared to those in naïve adults. They rarely present severe respiratory symptoms and often remain asymptomatic [3]. However, they can develop the life-threatening multisystem inflammatory syndrome MIS-C, while generally lacking severe respiratory symptoms [4,5,6,7]. The spike glycoprotein is the dominant exposed antigen on enveloped viruses and hence includes hot spots for mutations occurring in new variants, like Omicron lineages [8]. The spike protein includes the S1 subunit spanning the receptor binding domain (RBD), the N-terminal domain (NTD), and the more conserved S2 membrane fusion subunit [9,10], which are important targets for viral neutralization [11]. The spike can trigger a series of adaptive immune responses mediated by three major cell types: B cells (humoral immunity) and CD4+ and CD8+ T cells (cell-mediated immunity) [12,13]. While the RBD and NTD domains are exposed on the tip of S1 and are the target of most neutralizing antibodies [14], the less exposed S2 spike subunit is more conserved and includes cross-reactive epitopes with seasonal betacoronaviruses [15]. Pediatric individuals with previous SARS-CoV-2 infection generally exhibit higher titers of antibodies against the viral spike glycoprotein than adults [16,17]. Additionally, children under 5 years of age are found to have a great incidence of infection with the betacoronavirus HCoV-OC43, which is the common cold coronavirus that is most closely related to SARS-CoV-2, which may in part explain the high number of asymptomatic cases [15,18,19].

Wide vaccination coverage is a way to alleviate health systems, and an essential aspect to avoid collapsing medical care and to gain control over a pandemic. For COVID-19, cases continue to increase despite global vaccination campaigns [20,21]. Those include numerous safe and effective vaccines that have been developed to effectively reduce the risk of infection, severe disease, and death. Most vaccine strategies employ the spike protein as a target including the spike-coding vaccines based on mRNA BNT162b2 (Pfizer-BioNTech,) NY, USA; Mainz, Germany, mRNA-1273 (Moderna, Cambridge, MA, USA), and adenoviral vectors such as Ad26.COV2.S (Janssen-Johnson & Johnson, Beerse, Belguium; New Brunswick, NJ, USA), among others [22,23]. On the other hand, vaccines based on whole-inactivated viruses such as CoronaVac (Sinovac, Beijing, China) also include the spike protein in addition to other viral structural proteins such as the nucleoprotein [24]. Several studies have shown that antibody responses from the first massive vaccination against COVID-19 in early 2021 have decreased over the following six months after vaccination, likely contributing to an increase in breakthrough infections [25,26,27]. The emergence of numerous SARS-CoV-2 variants is one of the most important developments in the COVID-19 pandemic [28]. Yet, diverse variants of concern, interest and under-monitoring have different levels of increased transmissibility and resistance to existing immunity and have emerged sequentially [29]. Furthermore, these variants have been widespread and have evolved over time since the start of the pandemic [30,31,32].

To the best of our knowledge, the virological and immunological characteristics associated with the viral main lineages are key to informing health policies, including booster and vaccination schedules, and also informing the development of potential specific variants or pan-coronavirus vaccines. Important issues include whether the different variants escape vaccination-induced immune responses. In adults, mRNA-based vaccines recognize early variants of SARS-CoV-2, while significant overall decreases in humoral and cellular responses to Omicron lineages were recorded [33]. In addition, booster doses in adults induce neutralizing immunity against Omicron BA.1 infection [34]. A low effectiveness of the BNT162b2 and CoronaVac vaccines against several Omicron variants of SAR-CoV-2 has been described, reporting low levels of neutralizing antibodies in those vaccinated with BNT162b2 and loss of antibody reactivity in those vaccinated with CoronaVac [35,36].

Yet, scarce information on the immune responses in the pediatric population immunized with vaccination schemes based on CoronaVac and heterologous BTN162b2 booster doses is available [37,38]. It has been described that vaccination against COVID-19 in children aged 5–11 years showed lower effectiveness in preventing SARS-CoV-2 infection and severe COVID-19 than in individuals aged 12 years and older [39]. The CoronaVac vaccine has been associated with weaker neutralization titers against spike but yet comparable or higher T cell responses than mRNA and other vaccine platforms [40]. Clinical phase 1/2 studies with CoronaVac have shown that a two-immunization schedule (3 μg dose) induced humoral immune response and neutralizing antibodies in children and adolescents aged 3–17 years [41]. T-cell responses are elicited by the inactivated whole-virus vaccines, which are directed against all structural proteins of SARS-CoV-2 and not the spike protein alone. It has been further described that T cell responses against SARS-CoV-2 S, N, and M proteins are conserved for Omicron BA.1 [42]. Yet, vaccinated pediatric age groups are more susceptible to reinfections due to Omicron variants than previous variants [43]. However, it has been shown that three doses of BNT162b2 or three doses of CoronaVac were effective in preventing COVID-19, hospitalizations, and severe outcomes among the pediatric population during the Omicron-dominant pandemic [44,45]. Yet, more studies of pediatric cohorts are required to test their immune status against Omicron lineages. Also, more studies are needed to characterize the efficiency of heterologous vaccination in children and adolescents against SARS-CoV-2 Omicron variants. In this study, we analyzed which vaccination scheme, including CoronaVac (2x) alone, BNT122b2 (3x) alone or their heterologous immunization induces a higher humoral and cellular immune response against SARS-CoV-2 Omicron BA.1 variant in adolescents between 10 and 16 years. Our findings indicate that booster doses using BNT162b2 in pediatric individuals immunized before with CoronaVac (2x) exhibit an enhanced cellular and humoral immune response against Omicron BA.1 compared to CoronaVac (2x) alone.

## 2. Materials and Methods

### 2.1. Study Design and Population

We recruited teenager (10–16 years old) donors immunized with two doses of CoronaVac [CoronaVac (2x)], two doses of CoronaVac plus one dose of BNT162b2 [CoronaVac (2x) + BNT162b2 (1x)], two doses of CoronaVac plus two of BNT162b2 [CoronaVac (2x) + BNT162b2 (2x)], or three doses of BNT162b2 [(BNT162b2 (3x)]. It is important to note that these vaccination schemes were the most common vaccines used in Chile are CoronaVac (Sinovac, Beijing, China) and BNT162b2 (Pfizer, New York, NY, USA; BioNTech, Mainz, Germany), and the most common third booster shot was BNT162b2, in accordance with the booster vaccine policy implemented by the Chilean National Immunization Program (PNI) [46]. Despite being freely available, a considerable group of individuals in Chile has refused to receive booster doses.

The sample collection was performed in schools from the Metropolitan Region of Chile during the second semester of the year 2022 when the SARS-CoV-2 Omicron variants BA.1, BA.2, BA.4, BA.5 and BQ.1 were circulating predominantly [47]. The inclusion and exclusion criteria were applied to all groups of vaccinated teenagers aged 10 to 16, who must have the complete vaccination schedule against SARS-CoV-2 at least 30 days since their last immunization, with no history of previous SARS-CoV-2 infection. Also, the participants had no symptoms associated with COVID-19. It is important to note that these vaccination schemes were developed against the original strain of SARS-CoV-2 (Wuhan-Hu-1) when administered in Chile. The main inclusion criteria for donors were individuals between 10 and 16 years old, and no history of SARS-CoV-2 infection at least 8 months from the date of sampling. The main exclusion criteria included individuals under 10 or over 17 years old, pregnant women, individuals with morbid obesity, immunosuppression, or symptoms associated with COVID-19. Individuals who had a history of reinfection by COVID-19 were also excluded.

The vaccinated group included individuals who had completed their immunization schedule at least one month before sample collection and no more than 18 months before the collection. A total of 99 donors were included in the study. After applying a documentary filter, 88 donors were analyzed in this study; 18 received [CoronaVac (2x)], 35 received [CoronaVac (2x) + BNT162b2 (1x)], 21 received [CoronaVac (2x) + BNT162b2 (2x)], and 14 received triple BTN162b2 vaccination [BNT162b2 (3x)]. The timing of sample collection is described in Supplementary Table S1.

Samples were collected with authorization from the local ethics committee, and all participants and their respective parents or guardians provided informed consent by declaring and signing it.

### 2.2. Serum and PBMCs Preparation

Venous blood was used to obtain serum via a vacuum blood collection tube with clot activation and separating gel (BD Vacutainer) as described previously [48]. The tube was stored at room temperature for at least 30 min to allow the blood to clot and then centrifuged at 2000 rpm for 10 min at 4 °C; 2 mL of supernatant serum was recovered and liquated into two cryotubes at 1 mL each and stored at −20 °C until further analysis. Then, another two Vacutainer tubes (Heparin/Lithium) were used for the isolation of peripheral blood mononuclear cells (PBMC) by Ficoll gradient.

### 2.3. Antibody Detection

Immune response analysis was performed as described by Díaz-Dinamarca et al. [48] Immunoglobulin G (IgG) against SARS-CoV-2 nucleoprotein N (Snibe Diagnostic, Shenzhen, China, cat. 130219015M), receptor binding domain from the spike glycoprotein S1 (S1, Wuhan-Hu-1) (Snibe Diagnostic, cat. 130219017M) and hACE2-RBD inhibition assay method (Snibe Diagnostic, cat. 130219027M), all based on the ancestral Wuhan-Hu-1 SARS-CoV-2 sequences, were detected using a chemiluminescent immunoassay (CLIA) using commercial kits (Snibe). Briefly, 300 µL of serum was aliquoted into cryotubes and then analyzed using the Maglumi X8 CLIA detector according to the manufacturer’s instructions.

Immunoglobulin A (IgA) was detected using the enzyme-linked immunosorbent assay (ELISA) commercial kit COVID-19 human IgA spike-RBD and nucleoprotein of SARS-CoV-2 from Raybiotech (Cat: IEQ-CoVSN-IgA-1), following the manufacturer’s instructions. The samples were measured using a plate reader (EPOCH 2, Biotek, Charlotte, VT, USA).

### 2.4. Generation of rVSV-SARS2-S-BA.1

Recombinant vesicular stomatitis virus carrying the spike protein of SARS-CoV-2 (rVSV-SARS2-S) has been shown to allow for rapid quantification and correlate well in viral neutralization with convalescent serum compared to the authentic SARS-CoV-2 [49,50,51]. Given that this virus contains the spike of the ancestral Wuhan-Hu-1 SARS-CoV-2 strain, we prepared rVSV-SARS2-S containing the spike of the Omicron BA.1 variant. Therefore, the SARS-CoV-2 genome sequences corresponding to the sequencing of samples from individuals infected with the Omicron BA.1 variant in different countries were obtained (GISAID (Munich, Germany) accession ID: EPI_ISL_7373598; EPI_ISL_7373061; EPI_ISL_7371749; EPI_ISL_7370181; EPI_ISL_7368223; EPI_ISL_7358093; EPI_ISL_7358079; EPI_ISL_7358076; EPI_ISL_7358069; EPI_ISL_7356256; EPI_ISL_7355546; EPI_ISL_7354594; EPI_ISL_7352907; EPI_ISL_7350365; EPI_ISL_7350042; EPI_ISL_7349764; EPI_ISL_7348417; EPI_ISL_7346862; EPI_ISL_7345322; EPI_ISL_7337515), performing multiple alignments of the open reading frames corresponding to spike glycoprotein in the Clustal W program (Dublin, Ireland) and subsequently, the generation of the consensus sequence with the EMBOSS Cons tool (Hinxton, England) [52] which was compared with the sequence previously described for omicron BA.1 [53] to confirm the characteristic mutations of the variant. The consensus sequence was next synthesized (GenScript, Queenstown, Singapore) and cloned into the VSV-SARS-CoV-2 spike antigenome plasmid [49] (kindly provided by Dr. Kartik Chandran, Albert Einstein College of Medicine, NY, USA), replacing the sequence of the Wuhan-Hu-1 spike. This plasmid also encodes an eGFP reporter gene as an independent transcriptional unit. Recombinant virus rescue was carried out as previously described [49]. Supernatants from transfected cells were transferred to Vero E6 hACE2 cells (previously generated [50]) every day until the appearance of eGFP-positive cells examined under an inverted microscope (IX71; Olympus, Tokyo, Japan) and pictures taken (ProgRes C5; Jenoptik, Jena, Germany) for subsequent analyses (Supplementary Figure S1). RNA from the virus in infection passage 9 was sequenced (Macrogen, Singapore) to corroborate that the coding fragment corresponded to the spike of Omicron BA.1, including the additional non-silent mutations K145E y F372S, due to serial passaging. All experiments for the generation of rVSV-SARS2-S-BA.1 and the use of rVSV-SARS2-S viruses in the microneutralization assays were carried out at biosafety level 2.

### 2.5. rVSV-SARS2-S Microneutralization Assays

Microneutralization assays were performed with rVSV-SARS2-S as described previously [50]. Briefly, the serum samples were treated at 56 °C for 30 min prior to use. Serial sera dilutions were then incubated with rVSV-SARS2-S-Wuhan-Hu-1 [49], (kindly provided by Dr. Kartik Chandran, Albert Einstein College of Medicine, NY, USA) or rVSV-SARS2-S-BA.1 viruses (MOI of 0.25) for 1 h at 37 °C. The serum–virus inoculum was then added to Vero E6 hACE2 cells and infection was stopped by fixing cells with 4% formaldehyde (Pierce, Waltham, MA, USA) and stained for 5 min with 300 nM 4′,6-diamidino-2-phenylindole (DAPI) (Invitrogen, Waltham, MA, USA). Total fluorescence for GFP was measured on a Synergy plate reader (BioTek, Charlotte, VT, USA) (DAPI excitation at 360 nm and emission at 460 nm; GFP excitation at 485 nm and emission at 526 nm) and normalized against DAPI fluorescence. The half-maximal inhibitory concentration (IC50) of the sera was calculated using non-linear regression analysis based on data obtained from technical replicates.

### 2.6. Detection of T lymphocytes Activated against SARS-CoV-2 by Flow Cytometry

Activation-induced marker (AIM) detection was performed according to the method described by Grifoni et al., 2020 [54]. Briefly, PBMCs were thawed in RPMI-1640 medium supplemented with 5% human serum, penicillin/streptomycin, and L-glutamine. Subsequently, 1 × 10^6^ PBMCs were stimulated with SARS-CoV-2 variant peptide combinations (Wuhan-Hu-1 and Omicron BA.1; 1 μg/mL) in a 96-well U-bottom plate at 37 °C for 24 h. The peptides were overlapping 15-mers by 10 aminoacid spanning the spike protein corresponding to ancestral or BA.1 Omicron sublineage as previously described [33]. Cells were further stimulated with an equimolar concentration of DMSO (negative control). After stimulation, cells were analyzed by flow cytometry as described below. Then, cells were stained with the following antibodies: anti-CD69-PerCP, anti-CD4-V510, anti-CD8-allophycocyanin (APC)-H7, anti-CD134(OX-40)-phycoerythrin (PE)-Cy7, anti-CD137-BV421, anti-CCR7-fluorescein isothiocyanate, anti-CD45RA-PE. FVS660 was included in cell staining (1:1000, APC; BD) and samples were processed on FACSVerse (BD Biosciences, Franklin Lakes, NJ, USA). SARS-CoV-2-specific T cells were detected by co-expression of AIM on CD4+ (OX40 and CD137) or CD8+ (CD69 and CD137) T cells [54]. The activation markers CD4+ (OX40 and CD137) or CD8+ (CD69 and CD137) T cells were validated according to Grifoni et al. [54]. The DMSO-stimulated sample was used to establish the cutoff for the activation markers. In total, 30,000 events were acquired in the pool of live cells per sample.

### 2.7. Statistical Analysis

In this study, the geometric mean and 95% confidence interval (CI) were estimated for each group of immunized individuals. To compare whether there was a statistically significant difference between antibody titers, neutralizing antibodies, and AIM T cells from different vaccine combinations, an ANOVA test was used after the logarithmic transformation of antibody data and Kruskal–Wallis test for AIM T cells [55,56,57,58]. To assess the differences in responses between Omicron-BA.1 and Wuhan-Hu-1 within each scheme, a Wilcoxon test was applied.

If a statistically significant result was obtained from the ANOVA test, post hoc pairwise comparisons were performed using Tukey’s Honest Significant Difference (HSD) method, which uses the Studentized range distribution to estimate confidence intervals of factor level mean differences. For the Kruskal–Wallis test, Mann–Whitney was applied.

The fold-change (FC) was calculated as the Omicron.BA-1/Wuhan-Hu-1 ratio of T cells in individuals vaccinated with each scheme, and comparisons of the geometric mean values (GMV) of these fold-changes were conducted within and between different vaccination schemes. For this purpose, after logarithmic transformation of the FC, T-test (by T-test compared with a hypothetical mean of 1) and ANOVA with Tukey’s post-hoc were applied, respectively.

Statistical tests were evaluated with a 5% significance level. The statistical analyses and charts performed were obtained using R (v.4.2.3) and RStudio (v.2022.12.0.353).

## 3. Results

- The double booster with BNT162b2 generated an increase in humoral immunity against SARS-CoV-2

**Figure 1:**
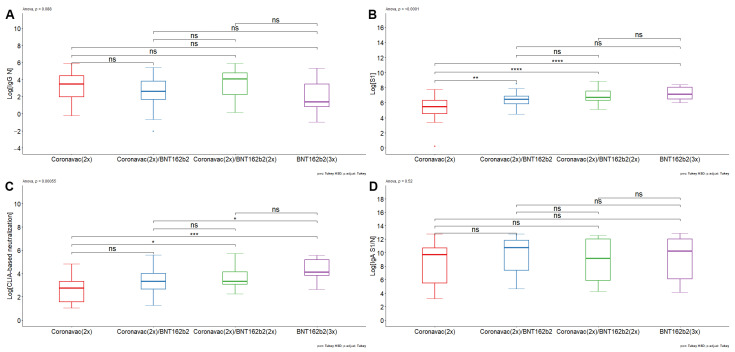
Comparison of antibody responses against different SARS-CoV-2 antigens in pediatric individuals immunized with different schemes of CoronaVac and BNT16b2 vaccines. CLIA analysis was used to measure serum IgG against the SARS-CoV-2 (**A**) nucleoprotein, (**B**) S1-RBD domain, and (**C**) ACE2-RBD inhibition assay. Serum IgA antibodies against (**D**) the sum of nucleoprotein and S1-RBD protein of SARS-CoV-2 were measured by ELISA. Data describe statistical analysis based on (**A**) Log [IgG Nucleoprotein], (**B**) Log [IgG S1-RBD], (**C**) Log [CLIA-based neutralization], and (**D**) Log [IgA S1/Nucleoprotein]. Statistically significant differences between groups were determined with one-way ANOVA. Significant pairwise differences were obtained by Tukey’s HSD post-hoc (* *p* < 0.05; ** *p* < 0.01; *** *p* < 0.001; **** *p* < 0.0001; ns: not statistically significant).

We decided to analyze the humoral immune response against different viral antigens. Therefore, we first quantified antibodies against SARS-CoV-2 nucleoprotein (N) (Figure 1A). The volunteers who had received different vaccination schemes had similar ranges in the titers of antibodies against the N protein from SARS-CoV-2. CoronaVac (2x) had a GMV of 24.58 A.U. (95% CI: 9.92–60.95), whereas [CoronaVac(2x) + BNT152b2 (1x)] and [CoronaVac(2x) + BNT152b2 (2x)] had GMV of 13.14 A.U. (95% CI: 7.09–24.34) and 33.50 A.U. (95% CI:13.22–84.88), respectively. For the [BNT152b2 (3x)] scheme, the GMV was 6.13 A.U. (95% CI: 1.31–28.68). The results suggest that the pediatric population analyzed has antibodies against the N protein of SARS-CoV-2. Given that the nucleoprotein of coronaviruses is highly conserved [59], the presence of anti-N reactivity in all vaccine groups may imply either cross-reactivity derived from previous infections with different coronaviruses or may be derived from previous asymptomatic infections with SARS-CoV-2.

Next, we analyzed antibodies against S1 (Wuhan-Hu-1). Contrary to the results of antibodies against nucleoprotein, there was a lower titer of antibodies against the S1 protein in the pediatric population immunized with [CoronaVac(2x)] compared with all formulations and boosters based on BNT162b2 (Figure 1B). The results indicate that the GMV of IgG against S1 concentration for individuals vaccinated with [CoronaVac (2x)] serum was 191.47 A.U. (95% CI: 82.92–442.14 A.U.), whereas groups vaccinated with [CoronaVac (2x) + BNT162b2 (1x)] and [CoronaVac (2x) + BNT162b2 (2x)] had a GMV of 612.20 A.U. (95% CI: 453.62– 826.22 A.U.) and 1000.51 A.U. (95% CI: 650.21– 1539.53 A.U.), respectively. The group of individuals who had received [BNT162b2 (3x)] had a GMV of 1299.91 A.U. (95% CI: 783.37–2157.04 A.U.). The ANOVA test yielded a statistically significant result (*p*-value < 0.0001), where pairwise comparisons showed statistically significant differences between [CoronaVac (2x)] and [CoronaVac (2x) + BNT162b2 (1x)] (*p*-value < 0.01), [CoronaVac (2x)] and [CoronaVac (2x) + BNT162b2 (2x)] (*p*-value < 0.0001), and BNT162b2 (3x) (*p*-value < 0.0001) (Figure 1B). There were no statistically significant differences between schemes comprising BNT162b2. The data suggest that BNT162b2-based formulations promote an increase in antibodies against S1 in the pediatric population.

Next, a CLIA-based hACE2 protein competition assay was performed as a correlation estimate of neutralizing antibodies against SARS-CoV-2 [48,60]. The [BNT162b2 (3x)] vaccination scheme generated a higher level of CLIA-based neutralization compared to the [CoronaVac (2x)] and [CoronaVac (2x) + BNT162b2 (1x)] scheme (Figure 1C). The results indicate that the GMV of CLIA-based neutralization antibodies for [CoronaVac (2x)] serum was 13.73 A.U. (95% CI: 7.62–24.73 A.U.), whereas the [CoronaVac (2x) + BNT162b2 (1x)] and [CoronaVac (2x) + BNT162b2 (2x)] had a GMV of 26.19 A.U. (95% CI: 18.10–37.89 A.U.) and 39.46 A.U. (95% CI: 25.57–60.91 A.U.), respectively. [BNT162b2 (3x)] had a GMV of 66.69 A.U. (95% CI: 36.84–120.74 A.U.) The ANOVA test yielded a statistically significant result (*p*-value < 0.001), where pairwise comparisons showed statistically significant differences between [CoronaVac (2x)] and [CoronaVac (2x) + BNT162b2 (2x)] (*p*-value < 0.05), [CoronaVac (2x)] and [BNT162b2 (3x)] (*p*-value < 0.001), and [CoronaVac (2x) + BNT162b2 (1x)] and [BNT162b2 (3x)] (*p*-value < 0.05) (Figure 1C). No differences were found between [CoronaVac (2x)] and [CoronaVac (2x) + BNT162b2 (1)]; [CoronaVac (2x) + BNT162b2 (2x)] and [BNT162b2 (3x)]; and [CoronaVac (2x) + BNT162b2 (1x)] and [CoronaVac (2x) + BNT162b2 (2x)]. The hACE2 protein competition data suggest that BNT162b2-based formulations promote an increase in CLIA-based neutralizing antibodies in pediatric individuals. Furthermore, the data indicate that the double booster with BNT162b2 leads to a rise in CLIA-based neutralizing antibodies against SARS-CoV-2, particularly beneficial for adolescents previously immunized with CoronaVac.

- Antibodies induced by CoronaVac- and BNT162b2-based vaccine schemes decrease their neutralizing efficacy against Omicron BA.1.

**Figure 2:**
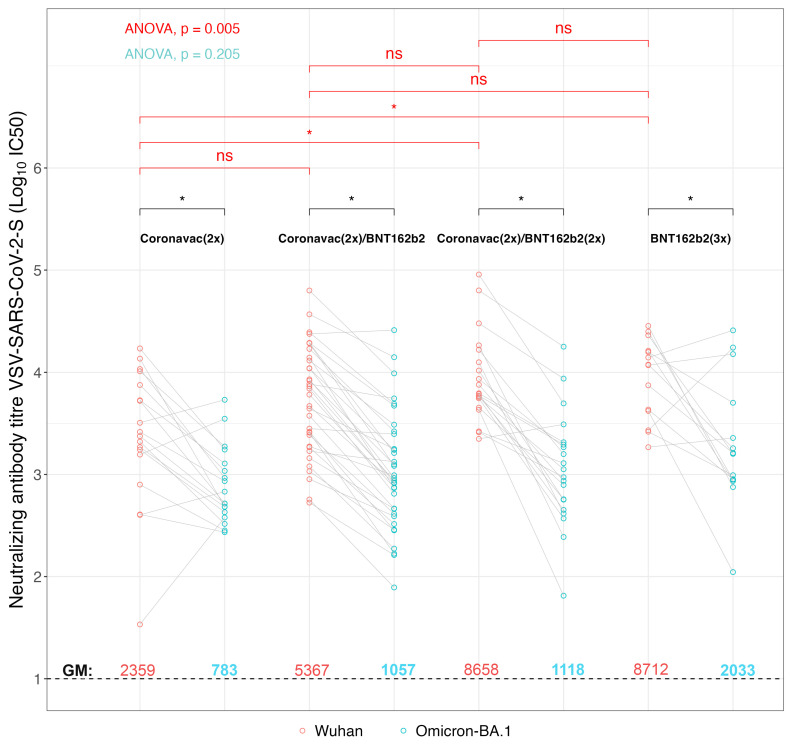
Neutralizing antibody responses against the ancestral SARS-CoV-2 Wuhan Hu-1 strain and omicron BA.1 variant in pediatric individuals immunized with different schemes of the CoronaVac and BNT162b2 vaccines. Serum-neutralizing antibody titers were determined by microneutralization assay of recombinant vesicular stomatitis virus carrying SARS-CoV-2 spike protein (rVSV-SARS2-S) from the Wuhan Hu-1 strain or the Omicron BA.1 variant. Data describe statistical analysis based on Log10 [IC50] titers. Dashed line indicates the limit of detection (LOD) of the microneutralization assay. Statistical significance within vaccination schemes was determined with T-test, and difference between vaccination schemes was determined with one-way ANOVA (*p* < 0.0001). Significant pairwise differences were obtained by Tukey’s HSD post-hoc (* *p* < 0.05; ns: not statistically significant).

Given the relevance of SARS-CoV-2 variants that were circulating during the sample collection, where Omicron lineages BA.1, BA.2, BA.4, BA.5 y BQ.1 were prevailing in Chile, we decided to analyze the pediatric samples in terms of the presence of neutralizing antibodies against the ancestral SARS-CoV-2 Wuhan-Hu-1 strain on which the vaccine formulations were based, and also against the Omicron BA.1 variant. For this, we used a viral system based on rVSV decorated with the SARS-CoV-2 spike from the Wuhan-Hu-1 or Omicron BA.1 strains (Figure 2). This viral system has been shown to correlate well with authentic SARS-CoV-2 in terms of viral cell entry and sera neutralization; at the same time, it does not require biocontainment measures and is easy to quantify [49,50,51]. The resulting virus neutralization data indicate that the GMV for the titrated IC50 were for [CoronaVac (2x)] 2358 IC50 (95% IC: 1100–5054 IC50) for the Wuhan-Hu-1 rVSV-SARS2-S virus and 782.52 IC50 (95% IC: 511–1196 IC50) for the rVSV-SARS2-S Omicron BA.1 virus, corresponding to a 3.01-fold decrease Wuhan-Hu-1/Omicron BA.1 in reactivity. In a similar way, the GMV [IC50] Wuhan-Hu-1/Omicron BA.1 was 5.07-fold decreased for the [CoronaVac (2x) + BNT162b2 (1x)] scheme, showing GMV [IC50] f 5367 IC50 (95% IC: 3493–8245 IC50) and 1057 IC50 (95% IC: 665–1680 IC50), for the Wuhan-Hu-1 and Omicron BA.1 rVSV-SARS2-S virus, respectively. The sera from pediatric individuals vaccinated with [CoronaVac (2x) + BNT162b2 (2x)] revealed a GMV [IC50] of neutralizing antibodies of 8658 IC50 (95% IC: 5442–13,774 IC50) and 1118 IC50 (95% IC: 616–2029 IC50), against the rVSV-SARS-2-S Wuhan-Hu-1 and Omicron BA.1 virus, respectively. Hence, while showing a high neutralizing antibody titer against the Wuhan-Hu-1 strain, the 7.74-fold decrease against the Omicron BA-1 strain generates GMVs that are similar to those observed with the vaccination group who had received only one booster dose of BNT162b2 [CoronaVac (2x) + BNT162b2 (1x)]. Finally, the group who had received [BNT162b2 (3x)] showed a 4.28-fold decrease in the GMV [IC50] neutralizing antibodies against Wuhan-Hu-1/Omicron BA.1 virus with a GMV of 8712 IC50 (95% IC: 5140–14,765 IC50) and 2032 IC50 (95% IC: 869–4750 IC50), respectively. Overall, all vaccination schedules had a decrease in neutralizing antibodies between Wuhan-Hu-1 and Omicron BA.1 rVSV-SARS-S virus (*p*-value < 0.01). It is important to note that despite the fact that this neutralizing antibody titer decreased in each vaccination group against the Omicron BA-1 strain Wuhan-Hu-1, the sera from some individuals of the different vaccination groups showed an increase. This may be indicative of an asymptomatic previous infection with one of the Omicron lineages. This is also in line with the observation that the IgG titers against nucleoprotein and IgA titers against S1/nucleoprotein did not show significant differences between the various immunization schemes.

An asymptomatic previous infection may also explain why no statistically significant difference was found between the different groups against the Omicron BA-1 strain, except for the group [CoronaVac (2x)] compared to the triple BNT162b2 [BNT162b2 (3x)] scheme (*p*-value < 0.05). When comparing the response against the Wuhan-Hu-1 strain, the [BNT162b2 (3x)] and [CoronaVac (2x) + BNT162b2 (2x)] generated a significant increase in neutralizing antibodies with respect to the [CoronaVac (2x)] group (*p*-value = 0.017), while the comparison of the neutralizing antibodies between [CoronaVac (2x)], and [CoronaVac (2x) + BNT162b2 (1x)], showed no significant differences. Together, these data suggest that the BNT162b2 double booster [CoronaVac (2x) + BNT162b2 (2x)], and the triple BNT162b2 vaccine [BNT162b2 (3x)], generated an increase in the protective humoral immune response.

- Vaccination schemes based on BNT162b2 improve cellular immune responses against Omicron BA.1. in pediatric population.

**Figure 3:**
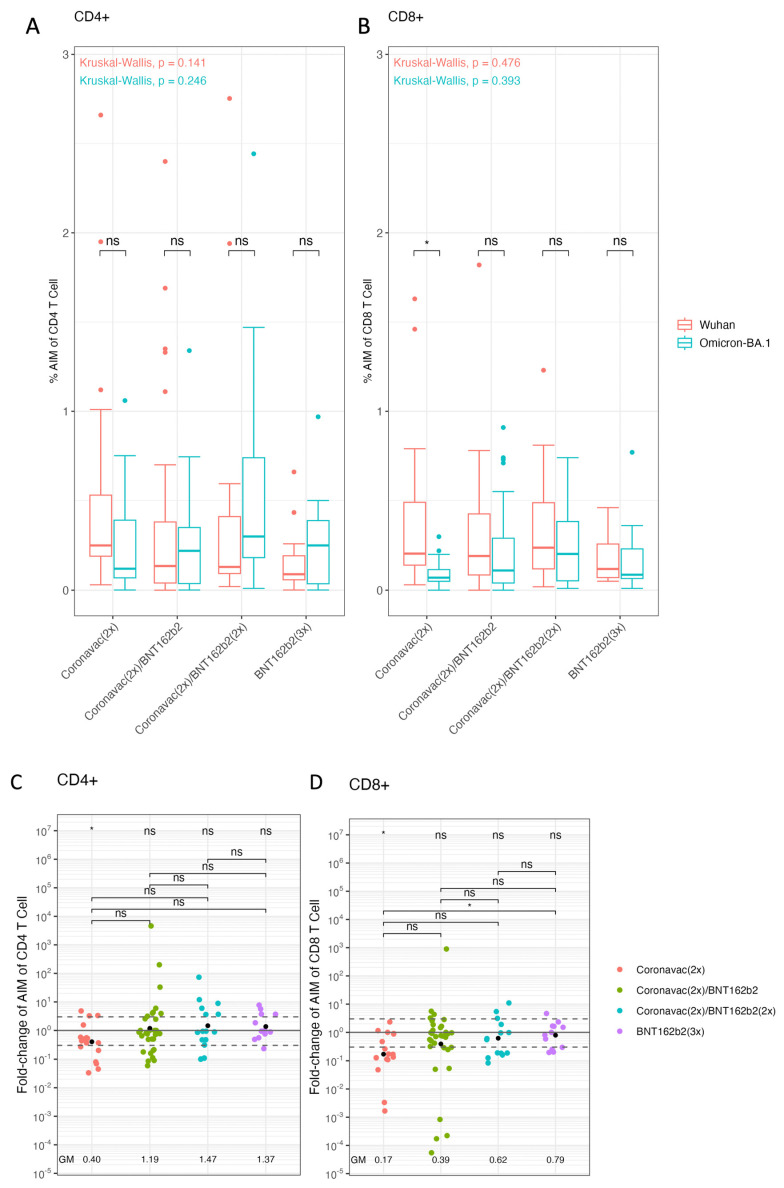
CD4+ and CD8+ T-cell responses against SARS-CoV-2 Wuhan Hu-1 strain and Omicron variant in pediatric individuals vaccinated with different schemes of CoronaVac and BNT162b2 vaccines. T cell activation was determined by AIM assay using a megapool of 15-mer peptides resembling the Wuhan Hu-1 or Omicron BA.1 spike proteins. The percentage of AIM of (**A**) CD4 T cells and (**B**) CD8 T cells was measured by flow cytometry. To assess the differences in responses between Omicron-BA.1 and Wuhan Hu-1 within each scheme, a Wilcoxon test was applied (* *p* < 0.05; ns: not statistically significant). A statistically significant difference between groups was determined with one-way Kruskal–Wallis (*p* < 0.05). Significant pairwise differences were obtained by Mann–Whitney post-hoc (* *p* < 0.05; ns: not statistically significant). Also, Fold-change (FC), between AIM+ Omicron.BA-1/AIM+ Wuhan (**C**) CD4 T cells and (**D**) CD8 T cells in the pediatric population were calculated. The figure displays the geometric mean (GM) for each vaccination scheme, which is represented by a black dot. A statistically significant difference between groups was determined with one-way ANOVA (*p* < 0.05). Significant pairwise differences were obtained by Tukey’s HSD post-hoc test (* *p* < 0.05; ns: not statistically significant).

The cellular immune response has previously been described to be of great importance against SARS-CoV-2 infection [61]. Despite the large number of spike protein mutations in the Omicron lineages, T-cell recognition is extensively cross-reactive against different SARS-CoV-2 variants [62]. We utilized T cell receptor (TCR) dependent AIM assays to identify and quantify spike-specific CD4+ T and CD8+ T cells in the pediatric population. AIM assay is a cytokine-independent method for the identification of Ag-specific T cells based on the increased expression of activation markers after Ag restimulation [63]. The T cell responses were analyzed by the activation of CD4+ T lymphocytes through Wuhan-Hu-1 or Omicron BA.1 strain peptide combinations (Figure 3A). The processed data showed that each vaccination scheme presented similar CD4+ T cell activation levels by Wuhan-Hu-1 and Omicron BA.1 spike peptides. In addition, when comparing vaccination schemes, there were no significant differences in activation for Wuhan-Hu-1 and Omicron BA.1. Then, the T cell response was analyzed in the activation of CD8+ T lymphocytes by Wuhan-Hu-1 or Omicron BA.1 variant peptide combinations for the different vaccination schemes (Figure 3B). Within the aforementioned tests, the [CoronaVac (2x)] vaccination scheme with spike peptides from Wuhan-Hu-1 versus Omicron BA.1 was the only one to show significant differences. In this case, Omicron BA.1 spike peptides generated less activation compared to Wuhan-Hu-1 peptides in the scheme based on [CoronaVac(2x)] (*p*-value = 0.004).

A more exhaustive analysis of the activation of T lymphocytes was performed by calculating a GMV fold-change for the activation of CD4+ and CD8+ T cells by Wuhan-Hu-1 and Omicron BA.1 peptides (Figure 3C,D). The resulting data indicate that the GMV of fold-change of CD4+ T cells AIM ratio (Omicron BA.1/Wuhan-Hu-1) for [CoronaVac (2x)] was as low as 0.401 (95% CI: 0.189–0.854), whereas the [CoronaVac (2x) + BNT162b2 (1x)], [CoronaVac (2x) + BNT162b2 (2x)] and [BNT162b2 (3x)] had a GMV fold-change of the AIM ratio of (Omicron BA.1/Wuhan-Hu-1) 1.186 (95% CI: 0.536–2.625), 1.466 (95% CI: 0.534–4.027), and 1.371 (95% CI: 0.688–2.732), respectively. T-test achieved statistically significant results showing a decrease in GMV Omicron BA.1/Wuhan-Hu-1 in [CoronaVac (2x)] (*p*-value < 0.01). The comparisons between schemes with the ANOVA test did not lead to significant differences (Figure 3C). Furthermore, the GMV of fold-change of AIM CD8+ T cells of Omicron BA.1/Wuhan-Hu-1 for CoronaVac (2x) was 0.168 (95% CI: 0.058- 0.486), whereas the [CoronaVac (2x) + BNT162b2 (1x)] and [CoronaVac (2x) + BNT162b2 (2x)] had a GMV of 0.388 (95% CI: 0.130–1.157) and 0.618 (95% CI: 0.256–1.487), respectively. In the [BNT162b2 (3x)] had a GMV of 0.791 (95% CI: 0.406–1.542). *T*-test provided statistically significant results between GMV fold-change of [CoronaVac (2x)] (*p*-value < 0.001). Between schemes an ANOVA test was applied showing significant differences (*p*-value = 0.0292) performing a Tukey post-hoc led to meaningful results between [CoronaVac (2x)] and [BNT162b2 (3x)] (*p*-value = 0.042) (Figure 3D). Our results suggest that the booster with BNT162b2 in the pediatric population vaccinated with [CoronaVac (2x)] improves the cellular immune response of CD4+ and CD8+ T lymphocytes against the Omicron spike.

## 4. Discussion

In this study, we characterized the humoral and cellular immune response against the Omicron variant BA.1 in adolescents attending schools in Santiago, Chile. Our work describes that in adolescents 10–17 years of age, the immunization with [BNT162b2 (3x)] or double BNT162b2 booster vaccination after two doses of CoronaVac [CoronaVac (2x) + BNT162b2 (2x)] promotes humoral immunity and cellular immune response against Omicron BA.1. Neutralizing antibody responses are crucial to reduce COVID-19 disease severity [64]. However, significant decreases were observed for memory B cells and neutralizing antibodies over time independently of the SARS-2 variants. In our study, we observed higher IC50 titers in the pediatric cohort compared to adults, which significantly decreased with the Omicron BA.1 strain compared to the ancestral virus. Interestingly, studies have shown that prior infection with SARS-CoV-2 can boost and broaden immunity related to neutralizing antibodies and T cell responses [33]. Regarding this point, the pediatric population characterized here had anti-nucleoprotein antibodies, and some individuals showed increased neutralizing antibody titers against Omicron BA.1 compared to the ancestral Wuhan-Hu-1 strain, coinciding with the period of sample collection and suggesting a previous SARS-CoV-2 infection independent from vaccination. Such a possible former infection would be asymptomatic according to the inclusion criteria of our study. Interestingly, children 3-to-5 years of age who had been vaccinated only with two doses of CoronaVac [CoronaVac (2x)] have been shown to be more susceptible to asymptomatic infections with SARS-CoV-2 [65]. Hence, in this cohort study, asymptomatic infections may have increased the neutralizing antibody titers independent of vaccination. In any case, even in the presence of possible previous infections, we show that the booster with BNT162b2 promotes anti-spike antibodies, increased neutralization and evokes immunity against Omicron BA.1 in the [CoronaVac (2x) + BNT162b2 (2x)] and [BNT162b2 (3x)] vaccination scheme groups. Such a heterologous prime-boost vaccination with CoronaVac followed by BNT162b2 has also been reported by others to induce high neutralizing titer against SARS-CoV-2 Omicron strains in the pediatric population [66].

SARS-CoV-2 vaccines of different platforms including mRNA, adenoviral vector and inactivated vaccines have been shown to produce T cell responses and have very high effectiveness against hospitalization [67,68]. mRNA-based vaccines have been reported to induce memory T lymphocytes that do not affect their response against other variants [33]. Furthermore, regardless of the AIM assay, responses against Omicron variants have been described to be highly conserved in CD4+ and CD8+ T cells [33,69]. In this context, CoronaVac can stimulate CD4+ T cell responses against spike peptides [69]. Our data presented here provide additional evidence that the booster with BNT162b2 generates an increase in the AIM assay of CD4+ and CD8+ T lymphocytes specific to Omicron BA.1 spike protein in the pediatric population immunized with [CoronaVac (2x)]. Among the different vaccination groups, we were able to detect significant differences for CD8+ T cell responses only between [CoronaVac (2x)] compared to [BNT162b2 (3x)]. However, we did not detect a pronounced T-cell immune enhancement by booster vaccination with BTN162b2 among the other immunization groups as described in previous studies [40,70,71]. This difference may be related to the AIM detection method that we used which relies on cytokine-independent measurement of T-cell activation based on a defined peptide pool based on SARS-CoV-2 spike T cell epitopes. Using these peptides, it was that CD4+ T cells in particular show cross-reactivity with spike peptides in 40–60% of SARS-CoV-2 unexposed individuals [56]. Such a T cell cross-reactivity may be even more pronounced in our patient cohort involving teenagers, who are at high exposure to common cold coronavirus infections. As a whole, while providing valuable new information, our study also had some limitations. One important limitation concerns our study cohort. At the time when we started recruiting our patient cohort, we were not able anymore to access samples of the pediatric population prior to the SARS-CoV-2 pandemic. Hence, we could not establish a vaccination control group as a baseline to differentiate SARS-COV-2-specific reactivity from cross-reactivity against structural proteins from other coronaviruses, particularly the N protein. By detecting anti-N antibodies, we hence cannot discard that the pediatric cohort may have developed immune responses by asymptomatic infections in addition to the vaccination schemes. This may explain why almost no cell-mediated immunity enhancement effect was observed by booster vaccination with BNT162b2. Further, our cohort showed a variation in the time intervals from the moment of the last booster dose until the sample acquisition among the different groups analyzed. Given the decay of immune responses over time, this variable may have further influenced the obtained results. On the other hand, we could not recruit a CoronaVac group (3x) because it is an uncommon scheme in the Chilean pediatric population. Another limitation of our study is generated by the study design. We only focused on the cellular immune response measured by AIM T cell responses detected only by CD4+ CD134(OX40)+ CD137+ and CD8+ CD69+ CD137+ markers; additional memory T lymphocyte profiles and/or markers such as IL-2 and IFN-γ could provide additional significant information in the comparison the different schemes against spike from Omicron BA.1 vs. Wuhan viruses. Finally, our study was only focused on the entire spike glycoprotein of SARS-CoV-2 and not against S1 and S2 subdomains separately or different epitopes within the RBD. Hence, no information on their neutralizing breadth can be provided as has been established elsewhere [72].

Finally, the findings obtained with our study cohort indicate that vaccination campaigns in the pediatric population implying booster doses, such as BNT162b2 and heterologous schemes, induce enhanced cellular and humoral immune responses against Omicron lineages. Currently, there is apprehension regarding the potential of SARS-CoV-2 emerging variants to evade the immune response elicited by approved vaccines. Vaccine boosters are essential to prevent viral immune evasion and severe forms of disease in the pediatric population.

## Data Availability

The original contributions presented in the study are included in the article/Supplementary Materials, further inquiries can be directed to the corresponding author/s.

## Supplementary Materials

The following supporting information can be downloaded at: https://www.mdpi.com/article/10.3390/vaccines12080919/s1, Figure S1: Generation of recombinant vesicular stomatitis virus decorated with the SARS-CoV-2 spike glycoprotein of the BA.1 omicron variant (rVSV-SARS-S-BA.1); Table S1: Timing of sample collection.
