# Supplementary material for: Booster Vaccination with BNT162b2 Improves Cellular and Humoral Immune Response in the Pediatric Population Immunized with CoronaVac
Source: Vaccines (Basel). 2024 Aug 15;12(8):919. doi: 10.3390/vaccines12080919 (PMC11359105; doi:10.3390/vaccines12080919)
Supplement: Supplementary file 1 [file vaccines-12-00919-s001.zip › vaccines-2887998-supplementary.pdf]

Supplementary Table S1: Timing of sample collection.

**Vaccine Scheme**	**N° of donors**	**Minimum time From last booster to sample collection** (months)	**Maximum time from last booster to sample collection** (months)	**Time range from last vaccine to sample collection** (mean; months)
CoronaVac (2x)	18	4.7	14.2	11.6
CoronaVac (2x)/ BNT162b2 (1x)	35	1.8	9.9	6.9
CoronaVac (2x)/ BNT162b2(2x)	21	1.8	6.0	3.4
BNT162b2 (3x)	14	2.4	11.5	7.3

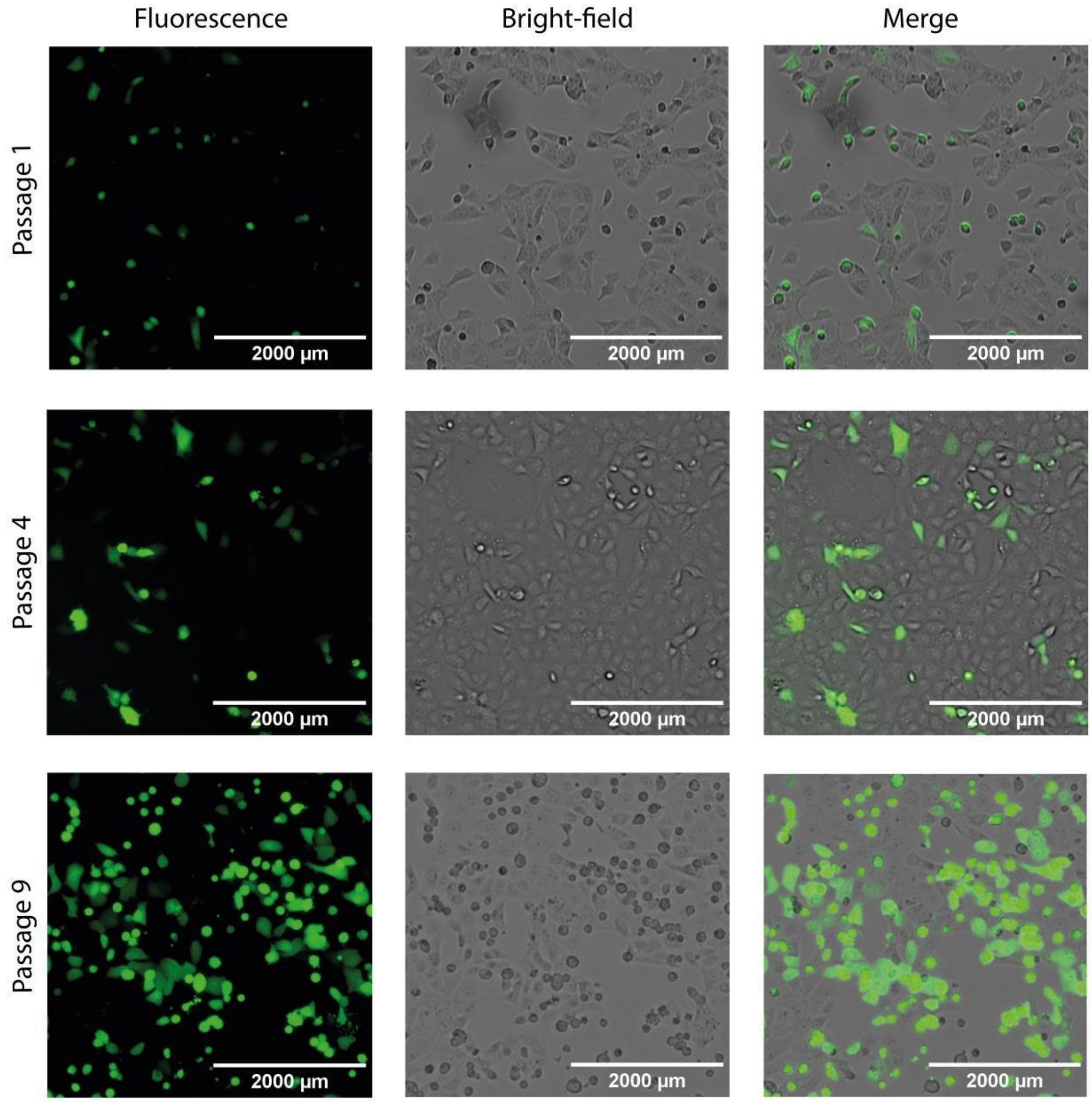

Figure S1. Generation of recombinant vesicular stomatitis virus decorated with the SARSCoV-2 spike glycoprotein of the BA.1 omicron variant (rVSV-SARS-S-BA.1). Formation of infectious focus in Vero E6 hACE2 cells previously infected with recombinant virus generated by transfected HEK293FT cells. Infection rounds 1, 4 and 9 are shown where the infected cells are seen in green fluorescence due to the expression of eGFP (left) in addition to the visualization of the cells in bright-field (middle) and merge (right) (scale bar, 2000 µm).
